# Advanced Chronic Rheumatic Heart Disease Associated With Substantial Myocardial and Valvular Fibrosis on Cardiovascular Magnetic Resonance

**DOI:** 10.7759/cureus.89991

**Published:** 2025-08-13

**Authors:** Olukayode O Aremu, Petronella Samuels, Stephen Jermy, Evelyn N Lumngwena, Daniel W Mutithu, Mary Familusi, Estelle Herbert, Aladdin Speelman, Sebastian Skatulla, Ntobeko A Ntusi

**Affiliations:** 1 Department of Medicine, University of Cape Town, Cape Town, ZAF; 2 Department of Medicine, Cape Heart Institute, University of Cape Town, Cape Town, ZAF; 3 Extramural Unit of Noncommunicable and Infectious Diseases, South African Medical Research Council, Cape Town, ZAF; 4 Cape Universities Body Imaging Centre, University of Cape Town, Cape Town, ZAF; 5 School of Clinical Medicine, University of the Witwatersrand, Johannesburg, Johannesburg, ZAF; 6 Department of Civil Engineering, University of Cape Town, Cape Town, ZAF; 7 Department of Civil Engineering, Centre for Research in Computational and Applied Mechanics (CERECAM), University of Cape Town, Cape Town, ZAF; 8 Department of Radiography, Cape Peninsula University of Technology, Cape Town, ZAF; 9 Extramural Unit on Intersection of Noncommunicable Diseases and Infectious Diseases, South African Medical Research Council, Cape Town, ZAF

**Keywords:** cardiovascular magnetic resonance, ecv, heart failure, lge, myocardial fibrosis, rheumatic heart disease, t1 mapping

## Abstract

Introduction: Rheumatic heart disease (RHD) is a major public health concern, particularly in low- and middle-income countries. Cardiovascular magnetic resonance (CMR) is a reliable noninvasive technique that detects fibrosis and inflammation in cardiovascular diseases. The study aimed to investigate the association of advanced chronic RHD with myocardial and valvular fibrosis using multiparametric CMR.

Methods: We assessed myocardial tissue characteristics in chronic RHD using multiparametric CMR. Forty-seven patients with severe, advanced RHD booked for valve replacement surgery, diagnosed on echocardiography, and matched with 30 healthy controls were scanned using a 3T MRI Siemens Magnetom Skyra scanner (Siemens, Erlangen, Germany). Baseline demographic and clinical characteristics, CMR findings including late gadolinium enhancement (LGE), T1 mapping, and extracellular volume (ECV) measurements were reported.

Results: The mean age of patients was 42 ± 12.8 years; 29 (62%) were women (39 ± 12.1 years). LGE CMR detected focal myocardial fibrosis in 47 (100%) of RHD patients vs. 2 (7%) of controls (p < 0.001). In all RHD patients, LGE involving all segments was observed. Patterns of LGE were linear 12 (26%), patchy 17 (36%), and diffuse 18 (38%). The total mass of myocardial enhancement with LGE imaging was significantly different from control (26 ± 10.1 vs. 17 ± 9 g, p = 0.002), resulting in a total percentage of (26% ± 6% vs. 21% ± 6%, p = 0.01). We also report elevated mean native T1 (p < 0.001) and ECV (p < 0.001) in all RHD patients compared to controls, respectively. Myocardial fibrosis is abundant in patients with severe, chronic RHD.

Conclusion: We report a high fibrotic burden in patients with advanced chronic RHD. Our observations may provide an explanation for increased heart failure and mortality in RHD, as excess myocardial fibrosis has been strongly linked with mortality in other disease contexts.

## Introduction

Rheumatic heart disease (RHD) follows acute rheumatic fever (ARF) and is a delayed sequela of an immunological response to Group A β-hemolytic streptococcal pharyngitis [1]. RHD is one of the three leading cardiovascular diseases (CVD) in sub-Saharan Africa [2]. ARF may result in valvulitis at the first episode [3], subsequently leading to established RHD with chronic valvular disease [4]. Sixty percent of ARF patients progress to chronic RHD after the first episode of ARF in the Northern Territory of Australia [2].

Late gadolinium enhancement (LGE), a sensitive and reproducible technique in cardiovascular magnetic resonance (CMR), remains the workhorse technique for myocardial characterization and evaluation of focal myocardial fibrosis [5]. Since LGE is based on extracellular space signal difference visualization in different areas of the myocardium, it may sometimes be difficult to identify diffuse myocardial fibrosis with LGE due to low contrast differentiation and inadequate signal nulling [6]. Parametric mapping, such as native T1 and extracellular volume fraction (ECV) mapping, is an accurate biomarker in many CVDs associated with diffuse fibrosis where mixed fibrotic patterns exist [7].

Myocardial ECV represents the percentage of tissue volume corresponding to the extracellular space, which increases in the presence of diffuse fibrosis [8]. ECV is effective in visualizing and quantifying both focal and diffuse myocardial fibrosis of ischemic or nonischemic etiology [9]. However, despite the distribution of gadolinium and different patterns of enhancement seen in other nonischemic CVD, information regarding the parametric assessment of myocardial fibrosis in RHD is yet to be well elucidated [6]. Therefore, we aimed to evaluate the presence of myocardial fibrosis using LGE, T1, and ECV measurement. We think that the study of myocardial fibrosis in RHD is important as it will inform an improved understanding of the mechanisms of heart failure and excess mortality in this condition.

This article was previously posted to the medRxiv preprint server on December 7, 2023.

## Materials and methods

Patient population

We provided a descriptive analysis of a prospectively designed study involving 47 patients echocardiographically confirmed to have chronic RHD, all booked for valve replacement surgery, and enrolled between August 2017 and August 2019, at the Cardiac Clinic, Groote Schuur Hospital, Cape Town. Patients with coronary heart disease, cardiomyopathy, concomitant congenital heart disease, hypertension, pericardial disease, any other valvular abnormality not due to RHD, and any contraindication for CMR, such as creatinine 2 g/dL, severe chronic kidney disease (glomerular filtration rate <30 mL/minute), metallic implant, pregnant, claustrophobic, and unable to lay still during the examination, were excluded from the study.

CMR protocol

Eligible participants were scanned with a 3T MRI Siemens Magnetom Skyra scanner (Siemens, Erlangen, Germany) with an 18-channel phased array body coil. Left ventricular (LV) volumes and masses were obtained during expiratory breath-hold for approximately 12 seconds and were prospectively electrocardiographically gated. LV volumes and mass were acquired using a standard CMR protocol (3T MRI Siemens Magnetom Skyra scanner). Steady-state free precession imaging (repetition time = 43.08 ms, echo time = 1.61 ms, flip angle = 40°, matrix size = 149 x 208, bandwidth = 962 Hz/px, slice = 8 mm thickness, 25 phases) was performed to obtain long-axis cines and a contiguous short-axis stack cines for assessment of LV volumes, mass, and ejection fraction, acquired over nine heartbeats/slice. LGE imaging was performed 10-15 minutes after gadolinium administration, and acquired using a short-axis stack, two-chamber, four-chamber, and LV outflow tract images to assess focal myocardial fibrosis. A standard dose of 0.2 mmol/kg of gadolinium diethylenetriaminepentaacetic acid (Magnevist, Bayer, South Africa) was administered intravenously in patients with moderate-to-mild renal function (estimated glomerular filtration rate > 30 mL/minute). Early gadolinium imaging was acquired in short stacks using a phase-contrast inversion recovery (PSIR) for the assessment of the presence of ventricular and left atrial appendage thrombus.

CMR image analysis

To analyze the LV volumes, including the LV end-diastolic volumes (LVEDV), LV end-systolic volumes (LVESV), LV myocardial mass (LVM), and LV ejection fraction (LVEF), the endocardial and epicardial contours of the LV were manually drawn from a stack of short-axis slices, excluding the papillary muscles on CVI42® software (Circle Cardiovascular Imaging, Calgary, Alberta). These parameters, except for LVEF, were indexed to the body surface area (BSA). Analysis was independently performed by two observers with at least three years of CMR experience.

Tissue characterization

The presence and extent of LGE were assessed by two readers with greater than five years of CMR experience and blinded to the diagnosis of participants. The assessment of cardiac function and chamber sizes was performed in standard views in the long-axis (horizontal and vertical) and short-axis planes. We recorded variables such as the presence or absence of LGE, the distribution patterns of LGE in different areas of the myocardium, the presence of valvular enhancement, and other findings. Both qualitative and quantitative assessments of the LGE were done. The quantitative LGE evaluation (both in grams and percentage of contoured myocardium) was calculated for each participant, utilizing a T1-weighted PSIR axial stack where the myocardium was contoured on a proprietary analytic software program (CVI42, Circle Cardiovascular Imaging, Calgary, AB), and a region considered to be normal myocardium was selected as a region of interest. Three standard deviations (SDs) were used as the threshold cut-off for the quantitative LGE (mean + 3 x SD). Patchy enhancement was defined as LGE enhancement that is focal, mid-myocardial, involving less than 50% of the diameter of the myocardium, and does not meet the traditional descriptions of linear, mid-wall, subendocardial, subepicardial, or transmural patterns of focal fibrosis. Diffuse enhancement was defined as a pattern of enhancement that is extensive, involving more than 50% of the myocardium, and often involving an entire segment, but is not truly transmural. LVEF was assessed with the following equation:

$\text{LVEF} = \frac{\text{LVEDV} - \text{LVESV}}{\text{LVEDV}}$.

For T1 and T2 mapping, CVI42® software was utilized to process images. Each ECV measurement was obtained by subtracting pre- and postcontrast values with hematocrit correction, usually obtained 15 minutes after the administration of contrast. The standard formula used was as follows:

$\text{ECV} = (1 - \text{hematocrit}) \times \frac{\left[ \frac{1}{T1_{\text{postcontrast}}(\text{myo})} - \frac{1}{T1_{\text{precontrast}}(\text{myo})} \right]}{\left[ \frac{1}{T1_{\text{postcontrast}}(\text{blood})} - \frac{1}{T1_{\text{precontrast}}(\text{blood})} \right]}$.

Statistical data analysis

Normality of data was tested using the Shapiro-Wilk normality test. Normally distributed data were presented as mean ± SD or, where highly skewed, as median (interquartile range); nonparametric data were presented as numbers (percentages). The chi-square test or the Mann-Whitney U test was utilized for nonparametric data. Unpaired samples between groups were assessed by the unpaired two-tailed Student t test. Correlation was assessed using Pearson’s R coefficient, as appropriate. All statistical tests were two-tailed, with p values of less than 0.05 considered statistically significant. All analyses were performed using Statistical Package for the Social Sciences version 25 (IBM Corp., Armonk, NY).

## Results

Patient population

Fifty-three RHD patients were recruited for the study, of whom 51 were scanned. Of the 51 patients scanned, three had incomplete scans due to difficulty with breath-hold (as they were in heart failure) and claustrophobia. One patient had findings consistent with a diagnosis of dilated cardiomyopathy and was excluded from the analysis. Thirty healthy controls were scanned for comparison: mean age = 39 ± 12.1 years, 16 (53%) were women. The remaining 47 patients were included in the study, 28 (64%) of whom were women. The mean age of the study population was 42 ± 12.8 years. As expected, there were no significant differences in height, weight, body mass index, and BSA compared to controls (Table 1). Just over half, 26 (55%), of patients with RHD reported symptoms of effort intolerance: New York Heart Association (NYHA) Class II, 12 (25%); NYHA Class III, 14 (30%).

**Table 1:** Demographic and clinical features of RHD patients and controls Continuous data are mean ± SD unless otherwise indicated. Characteristics are presented as 95% confidence interval BMI: body mass index; BSA: body surface area; RHD: rheumatic heart disease; SD: standard deviation

Parameters	Patients (n = 47)	Controls (n = 30)	p values (p < 0.05)
Age, years	42 ± 12.8	39 ± 12.3	0.28
Sex, n (%)			
Female	29 (62)	16 (53)	0.49
Male	18 (38)	14 (47)	0.49
Heart rate, bpm	82 ± 28	73 ± 15.9	0.07
Height, m	1.6 ± 0.1	1.7 ± 0.1	0.53
Weight, kg	77 ± 21.8	77 ± 19.4	0.96
BMI, kg/m^2^	28 ± 7.3	28 ± 5.7	0.68
BSA, m^2^	1.9 ± 0.3	1.9 ± 0.3	0.84

CMR functional and tissue characterization

RHD patients showed an increased indexed LVEDV, LVESV, LV stroke volume, and LVM compared to controls (all p < 0.001). LVEF was reduced in the patient cohort compared to controls (44.0% ± 13.3% vs. 57% ± 5.2%, p < 0001) (Table 2). There was significant left atrial dilatation in RHD patients compared to controls (41 ± 11.8 vs. 22 ± 3.13 mm, p = 0001). Increased indexed right ventricular (RV) end-diastolic volumes, RV end-systolic volumes, and RV stroke volumes were found. RV ejection fraction was significantly lower in RHD patients and below the normal range compared to controls (41% ± 15.9% vs. 54% ± 7.5%, p = 0.001). LGE was evident in all 47 (100%) patients with confirmed RHD, including 12 (26%), patchy 17 (36%), and diffuse 18 (38%) patterns of enhancement, but was only seen in two of the controls (7%).

**Table 2:** Functional CMR characteristics Continuous data are mean ± SD, unless otherwise indicated. Values are indexed to body surface area LVEDVI: left ventricular end-diastolic volume index; LVESVI: left ventricular end-systolic volume index; LVSVI: left ventricular systolic volume index; LVEF: left ventricular ejection fraction; LVMI: left ventricular mass index; LA: left atrium; RVEDVI: right ventricular end-diastolic volume index; RVESVI: right ventricular end-systolic volume index; RVSVI: right ventricular systolic volume index; RVEF: right ventricular ejection fraction

Parameters	Patients (n = 47)	Controls (n = 30)	p value (p < 0.05)
LVEDVI, mL/m^2^	113 ± 34.8	74 ± 14	<0.001
LVESVI, mL/m^2^	55 ± 18.8	32 ± 8.4	<0.001
LVSVI, mL/m^2^	46 ± 18.7	42 ± 7.5	<0.001
LVEF, %	45 ± 12.5	57 ± 5.2	<0.001
LVMI, g/m^2^	60 ± 30.7	32 ± 8.4	<0.001
LA diameter, mm	42 ± 12.3	22 ± 3.1	0.001
RVEDVI, mL/m^2^	77 ± 24.8	74 ± 13.5	0.31
RVESVI, mL/m^2^	47 ± 21.7	34 ± 9.6	0.01
RVSVI, mL/m^2^	33 ± 14.2	39 ± 7.3	0.04
RVEF, %	41 ± 15.9	54 ± 7.5	0.001

**Figure 1:**
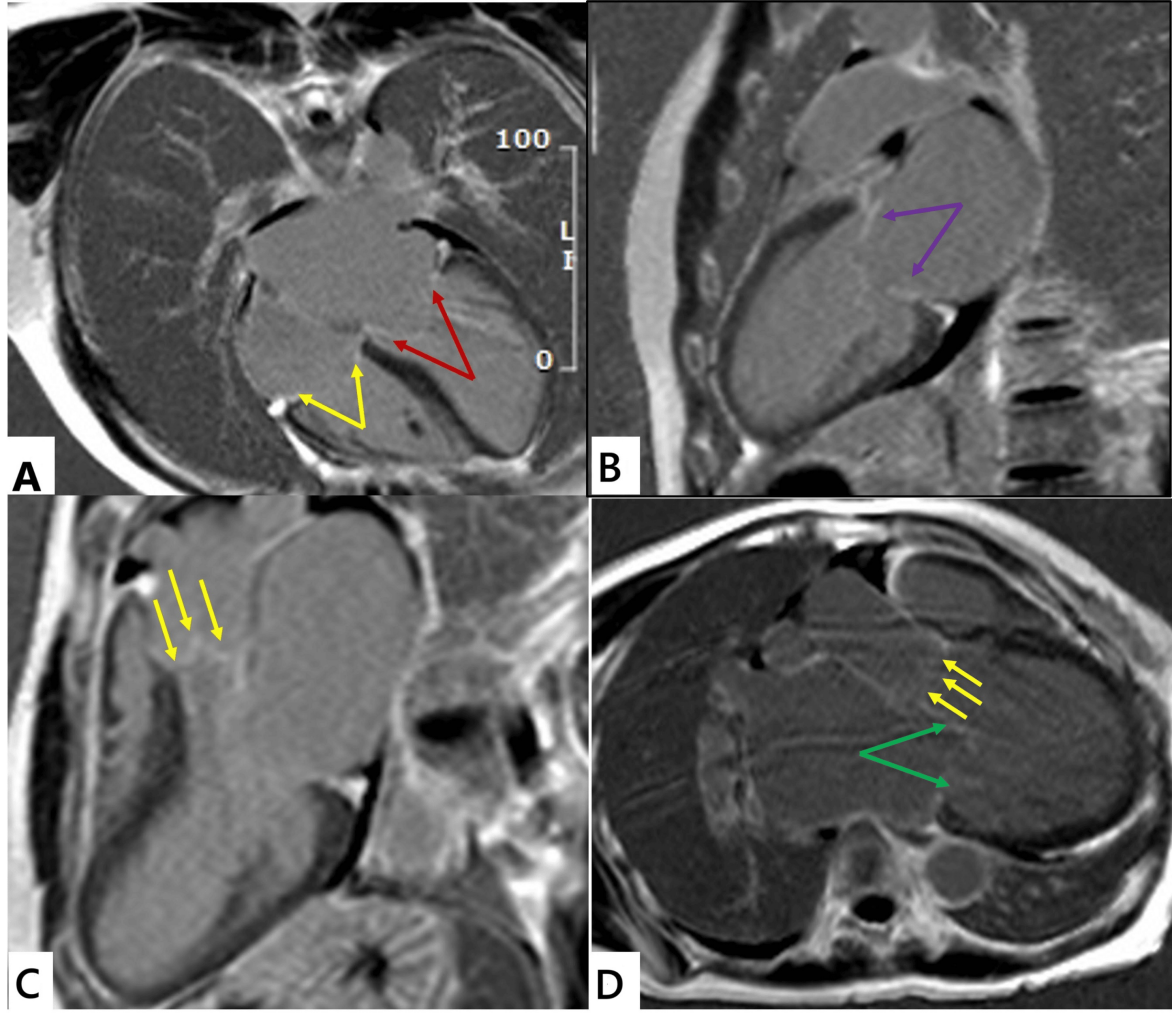
Valvular enhancement in RHD (A) A four-chamber view showing mitral and tricuspid valve enhancement indicated by red and yellow arrows, respectively. (B) A two-chamber view showing mitral valve enhancement indicated by purple arrows. (C) A three-chamber view showing aortic valve enhancement indicated by yellow arrows. (D) A three-chamber view showing aortic and mitral valve enhancement indicated by yellow and green arrows, respectively. Small pericardial effusions were noted frequently in RHD patients RHD: rheumatic heart disease

LGE of the mitral valve was noted in 31 (66%) of RHD patients, aortic valve enhancement was seen in two (7%) RHD patients, and no valve LGE was seen in controls (Figure 1).

In this study, LGE was observed in the atrial walls of 34 (73%) of RHD patients: left atrial (LA) and right atrial (RA) 24 (50%), LA 11 (23%), and RA 9 (2%). Small, subcentimeter pericardial effusions were seen in 43 (98%), and pleural effusions were found in three (6%) of patients with RHD, with none noted in controls (Table 3). Prominent myocardial crypts were observed in one RHD patient and in none of the controls.

**Table 3:** LGE, T1, ECV, and another finding Continuous data are mean ± SD unless otherwise indicated Values are presented as mean ± SD ECV: extracellular volume; LGE: late gadolinium enhancement; SI: signal intensity; SD: standard deviation; STIR: short tau inversion recovery

Parameters	Patients (n = 47)	Controls (n = 30)	p value (<0.05)
Presence of LGE, n (%)	47 (100)	2 (7)	<0.001
LGE mass, g	26 ± 10.1	17 ± 9	0.002
LGE percentage, %	26 ± 6	21 ± 6	0.01
Valvular enhancement, n (%)	32 (68)	0	0
Myocardial T2 STIR SI ratio, %	1.3 ± 0.3	1.5 ± 0.2	0.02
T1 value, ms	1,280 ± 55.9	1,213 ± 33.3	0.004
T2 value, ms	39 ± 2.9	39 ± 2.2	0.93
ECV, %	36 ± 0.05	28 ± 0.01	<0.001
Pericardial effusion, n (%)	46 (98)	0	0
Pleural effusion, n (%)	3 (7)	0	0
Crypts, n (%)	1 (2)	0	0

**Figure 2:**
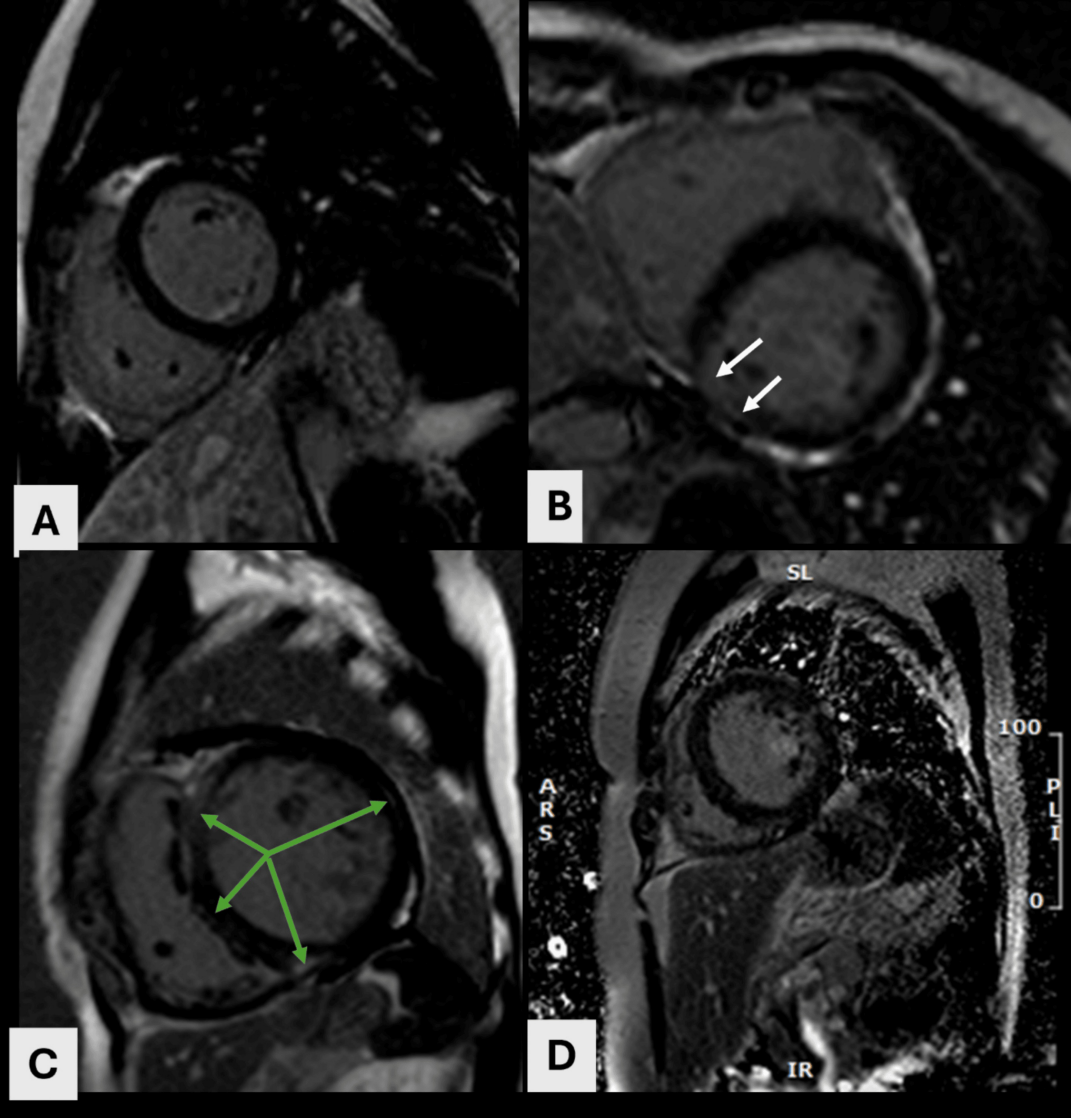
Patterns of LGE in RHD (A) Healthy control with no LGE. (B) Patchy mid-wall enhancement in all segments of the LV myocardium (white arrows). (C) Linear mid-wall enhancement in the anterior and mid septum, inferior LV-RV junction, and in the lateral wall. Also, note the small pericardial effusion. (D) Diffuse enhancement involving the entire myocardium LGE: late gadolinium enhancement; RHD: rheumatic heart disease; LV: left ventricular; RV: right ventricular

**Figure 3:**
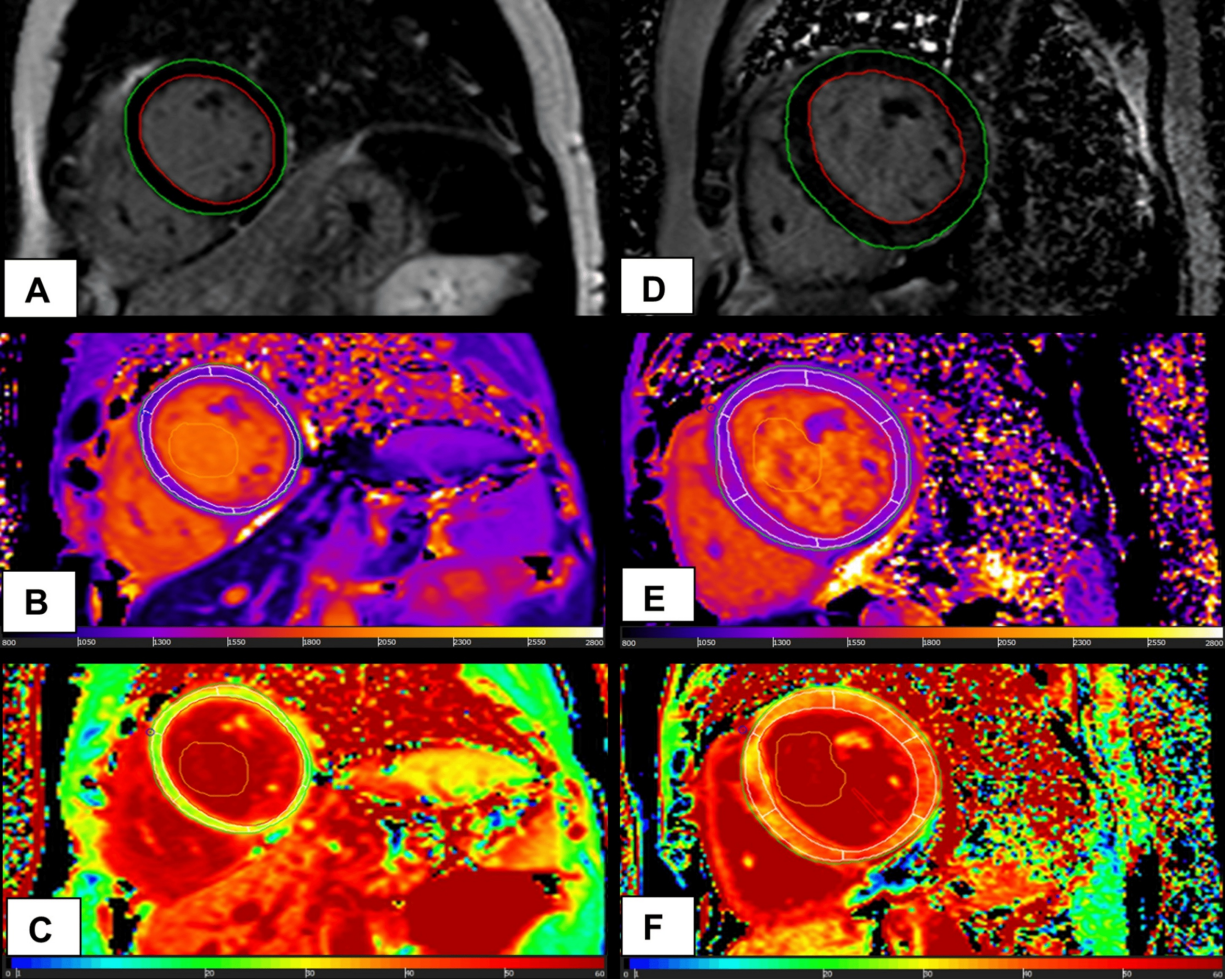
Native T1 and ECV is elevated in patients with RHD Tissue characterization at mid-ventricular slice in (A) b-SSFP cine image of the RHD patient (LVEF 52%); (B) native T1 value in RHD patient of 1,348 ms; and (C) postcontrast T1 imaging with ECV of 42%. (D) b-SSFP cine of a control (LVEF 59%); (E) native T1 value in control of 1,183 ms; and (F) ECV of 28%. Note the markedly elevated native T1 and ECV in RHD, even with minimal LGE ECV: extracellular volume; RHD: rheumatic heart disease; b-SSFP: balanced steady-state free precession; LVEF: left ventricular ejection fraction; LGE: late gadolinium enhancement

LGE was noted in 47 (100%) of RHD patients and two (7%) of controls (Figure 2). The total mass of myocardial enhancement with LGE imaging was significantly different from control (26 ± 10.1 vs. 17 ± 9.0 g, p = 0.002), resulting in a total percentage of 26% ± 6% vs. 21% ± 6.0%, p = 0.01). Mean native T1 values were elevated in all patients (1,280 ± 55.9 vs. 1,213 ± 33.3 ms in controls, p = 0.004) (Table 3 and Figure 3).

**Figure 4:**
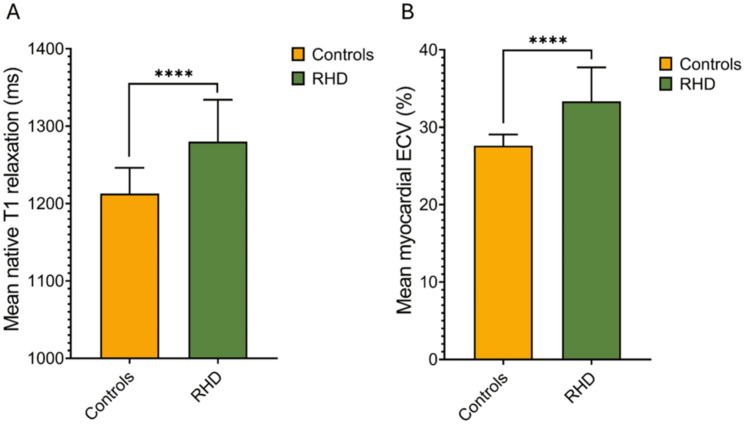
Tissue characteristics of RHD patients and controls Bar graphs showing elevated mean native T1 and ECV values in RHD patients (green bars) compared with healthy controls (orange bars). (A) Mean native T1 relaxation times were significantly higher in RHD patients compared to controls, indicating greater myocardial tissue alteration or fibrosis (****p < 0.0001). (B) Mean myocardial ECV percentage was also significantly elevated in RHD patients relative to controls (****p < 0.0001), further supporting the presence of increased diffuse myocardial fibrosis in RHD RHD: rheumatic heart disease; ECV: extracellular volume

ECV was elevated in patients and significantly higher compared to controls (36% ± 0.05% vs. 28% ± 0.01%, p < 0.001) (Figure 4). T2 values in RHD patients and controls were 39 ± 2.9 and 39 ± 2.2 ms, respectively, indicating the absence of myocardial inflammation in this chronic RHD cohort with advanced disease. Similarly, the T2 signal intensity ratio was within the normal range, corroborating the absence of myocardial inflammation.

## Discussion

CMR is the gold-standard imaging modality for the investigation of various CVDs of different etiologies [9]. It allows comprehensive characterization of functional, morphological, metabolic, tissue, and hemodynamic outcomes of cardiovascular pathologies [10]. We report on the multiparametric assessment of LGE and mapping in chronic RHD. In this descriptive study, patients with advanced chronic RHD were assessed with CMR, and LGE was detected in 47 (100%) of RHD participants. In addition, native T1 and ECV were elevated in RHD patients, supporting the presence of focal and diffuse fibrosis, whereas T2 values were normal in both controls and RHD patients, indicating the absence of acute inflammation. LGE of the mitral valve was noted in 31 (66%) of RHD patients. Mitral regurgitation was common and associated with dilation of the LA in most RHD patients, who also showed increased LVEDV, LVESV, and reduced LVEF.

RHD is the most common pathological cause of valvular heart disease in children and young adults, especially in low- to middle-income countries [11]. Mitral valve regurgitation is the most prevalent valve lesion in RHD, followed by mitral stenosis (MS), aortic regurgitation, and aortic stenosis [12]. Chronic RHD has a phenotype of subclinical inflammation and diffuse myocardial fibrosis that is a consequence of recrudescence and episodic tissue inflammation [13]. Several inflammatory mediators that attract systemic proinflammatory cytokine and soluble mediators are involved in the inflammatory process mediating RHD valvular regurgitation and/or stenosis, leading to local inflammation and tissue injury with subsequent cellular matrix disorganization, resulting in valvular dysfunction [14].

LGE imaging uses the principle of relative difference in volume of distribution of intravenously administered gadolinium (and subsequent variation of longitudinal relaxation, T1, times) between normal and abnormal myocardium [10]. LGE imaging is a sensitive, specific, and reproducible method used in assessing focal myocardial fibrosis and predicting prognosis in patients with ischemic and nonischemic cardiomyopathies [15]. In this study, LGE was evident in all the patients with confirmed RHD. Observed patterns of enhancement were linear 12 (26%), patchy 17 (36%), and diffuse 18 (38%) vs. 2 (7%) in controls. Contrastingly, Meel et al. reported LGE in four (18%) of their patient cohort, which might be due to the smaller sample size that they had, as well as more moderate disease in their cohort [16]. RHD is a chronic valvular disease caused by heart valve damage, resulting in severe or recurrent episodes of ARF [11].

A recent publication including 40 rheumatic MS patients from India, China, and Mexico reported that MS was associated with impairment in LV and RV systolic function, larger left and right atria, and LGE present in 32 (82%) of participants, similar to our study [17]. Shriki et al. found LGE in the atrial walls of three patients with chronic RHD [18]. In this study, LGE was observed in the atrial walls of 34 (73%) of RHD patients. We also observed RV free wall enhancement in one patient, in addition to valvular enhancement seen in the mitral valve in 31 (66%) patients, the aortic valve enhancement in 24 (5%) patients, and the tricuspid valve in nine (2%) patients. RHD predominantly affects the mitral valve, while aortic valve involvement is observed in 20%-30% of cases, and the tricuspid valve may be affected less commonly [1]. Multivalvular involvement and different enhancement patterns observed in this study could be due to the recurrent inflammation resulting in repeated endothelial and myocardial injury, leading to the fibrosis seen in chronic RHD [10].

T1 mapping has shown superiority in detecting diffuse myocardial structural changes, which may be difficult to quantify with LGE [19]. This is attributed to the fact that LGE relies on differences in signal intensity between healthy and diseased myocardium, whereas T1 mapping is a pixel-wise illustration of absolute T1 relaxation times on a map that circumvents the influence of windowing and nulling (as in LGE), allowing direct T1 quantification. As such, LGE is a reliable method to determine focal fibrosis [20]. However, in the presence of diffuse myocardial fibrosis, LGE becomes difficult to interpret as there is no healthy myocardium to use as a reference [5].

In this study, mean native T1 values were elevated in RHD patients, indicative of myocardial fibrosis. Fibrosis based on elevated native T1 values is further corroborated by the ECV and LGE findings in our patient cohort. Many studies have found a significant increase in myocardial T1 values in CVDs with diffuse myocardial fibrosis compared to healthy control participants [20-22]. Assessment of ECV shows great potential for being a noninvasive surrogate parameter for quantifying myocardial fibrosis and in the prediction of short-term mortality [23]. ECV is a derivative of T1 relaxation times, and it is calculated as the ratio of T1 change in the blood and myocardium, considering the hematocrit level, as previously explained [19].

Myocardial fibrosis in ARF is a consequence of inflammation following an immunological cascade triggered by cross-reactivity. This molecular mimicry causes an upregulation of autoantibodies, thereby exacerbating inflammation [24,25]. Myocardial inflammation and myocarditis in ARF may result in myocardial necrosis with subsequent fibrosis; myocardial fibrosis is prevalent in RHD and associated with adverse cardiovascular outcomes [26]. Myocardial extracellular matrix (ECM), which contains various proteins and signaling molecules maintaining cardiac structural integrity, provides a connection between intracellular cytoskeletal and intercellular proteins, which allows the heart to transmit biochemical signals, thereby activating the myofibroblasts [27]. Activation and differentiation of these myofibroblasts synthesize collagen I and III, which are later deposited in the ECM. In fibrosis, collagen I, which provides rigidity, overshoots collagen III, which provides elasticity [28]. CMR-LGE imaging with excessively retained gadolinium-based contrast agents represents a noninvasive standard for assessing myocardial viability and fibrosis because extracellular space is enlarged by dead CMs and postinfarct fibrosis [29].

Taken together, we used a multiparametric CMR approach to assess myocardial and valvular characteristics and observed evidence of myocardial fibrosis on LGE imaging in all patients with LGE enhancement that is focal and mid-myocardial, involving <50% of the diameter of the myocardium, and does not meet the traditional descriptions of linear, mid-wall, subendocardial, subepicardial, or transmural patterns of focal fibrosis. Additionally, we observed diffuse enhancement that was extensive, involving more than 50% of the myocardium and often affecting an entire segment, but it is not truly transmural. However, some patients with RHD exhibited both patchy and diffuse patterns of enhancement. Similarly, enhancement of the valves was common, typically affecting the mitral and aortic valves. Further, we observed significant differences in LV functional parameters, with a great reduction of LVEF in our RHD cohort. Native T1 and ECV values were elevated in RHD, which indicated prevalent myocardial fibrosis in all patients.

A limitation of this study was the modest sample size of 47, still making it one of the largest CMR studies of RHD. Our patients with severe advanced RHD probably are not representative of the general RHD population, as these were patients with long-standing disease who were awaiting valve replacement surgery, likely explaining the large burden of fibrosis. However, despite the small sample size, big differences were observed between RHD and demographically matched controls. The selection of advanced pathology will have introduced a degree of selection bias in the severity of phenotypes observed, which would have been avoided by including subjects representing the entire clinical spectrum of RHD. We observed a slightly elevated LGE percentage in the healthy controls. In a study conducted by Tahir et al., it was noted that the LGE mass/percent ranged between 0% and 1% [30]. However, many other studies have reported quantitative LGE to be up to 5%-10% in healthy controls. In our environment, where there is a high burden of tuberculosis and other viral infections, a higher burden of myocardial fibrosis from prior inflammation/infection is not unusual. Additionally, other factors that impact the burden of LGE in healthy controls include strenuous exercise and prior undiagnosed myocarditis. Comorbid conditions like hypertension in some of the controls may also have contributed to the slightly higher burden of LGE. In this study, we focused on the radiological aspect of the patient data rather than the clinical. Hence, we could not make any clinical correlations. Finally, there is a paucity of literature on CMR in RHD to draw comparisons from, and the existing studies have small sample sizes.

Implications for future study

The present study highlights a significant burden of myocardial and valvular fibrosis in patients with advanced chronic RHD, as demonstrated by multiparametric CMR. These findings emphasize the need for future research to explore the prognostic relevance of myocardial fibrosis in RHD, particularly its role in heart failure progression and mortality. Longitudinal studies are needed to assess how fibrotic burden evolves over time and whether it can serve as a biomarker for disease severity, treatment response, or surgical outcomes. Furthermore, large studies including patients at varying stages of RHD are essential to determine whether early myocardial fibrosis precedes overt valvular dysfunction and could be a target for early therapeutic intervention. Integration of clinical data, serological markers, and imaging findings may also help to elucidate the pathophysiological mechanisms linking chronic inflammation, autoimmunity, and myocardial remodeling in RHD.

## Conclusions

We report on the first multiparametric assessment of LGE and parametric mapping in severe chronic RHD with findings of LGE detected in 47 (100%) of RHD patients, who similarly demonstrated elevated native T1 and ECV, indicating presence of focal and diffuse fibrosis. T2 values were normal in both controls and RHD patients, indicating absence of acute and active inflammation. LGE of the mitral valve and atrial walls was common. These data, showing a high fibrotic burden, may provide an explanation for increased heart failure and mortality in RHD as excess myocardial fibrosis has been strongly linked with mortality in other disease contexts. This study shows that RHD patients of these population have a high fibrotic burden. New studies are needed to show if this high fibrotic burden could explain the heart failure and mortality in this patients, as excess myocardial fibrosis has been strongly linked with mortality in other disease contexts.
